# The Effect of Fragaria vesca Extract on Smear Layer Removal: A Scanning Electron Microscopic Evaluation

**DOI:** 10.7508/iej.2015.03.012

**Published:** 2015-07-01

**Authors:** Amin Davoudi, Sayed Alireza Razavi, Mohammad Hossein Mosaddeghmehrjardi, Mehdi Tabrizizadeh

**Affiliations:** a *Dental Students Research Center, Dental School, Isfahan University of Medical Sciences, Isfahan, Iran; *; b *Department of Endodontics, Gorgan University of Medical Sciences, Gorgan, Iran;*; c * Department of Pharmacology, Dental School, Shahid Sadoughi University of Medical Sciences, Yazd, Iran; *; d *Department of Endodontics, Dental School, Shahid Sadoughi University of Medical Sciences, Yazd, Iran*

**Keywords:** Fragaria vesca, Irrigation, Scanning Electron Microscopy, Smear Layer

## Abstract

**Introduction:**

Successful endodontic treatment depends on elimination of the microorganisms through chemomechanical debridement. The aim of this *in vitro *study was to evaluate the effectiveness of Fragaria vesca (wild strawberry) extract (FVE) on the removal of smear layer (SL).

**Methods and Materials:**

In this analytical-observational study, 40 extracted mandibular and maxillary human teeth were selected. After canal preparation with standard step-back technique, the teeth were randomly divided into 4 groups according to the irrigation solution: saline (negative control), 5.25% NaOCl+EDTA (positive control), FVE and FVE+EDTA. The teeth were split longitudinally so that scanning electron microscopy (SEM) photomicrographs could be taken to evaluate the amount of remnant SL in coronal, middle and apical thirds. The data were analyzed statistically by the Kruskal-Wallis and Mann Whitney U tests and the level of significance was set at 0.05.

**Results:**

Significant differences were found among the groups (*P*<0.001). The use of NaOCl+EDTA was the most effective regimen for removing the SL followed by FVE+EDTA. FVE alone was significantly more effective than saline (*P*<0.001).

**Conclusion:**

FVE with and without EDTA could effectively remove the smear layer; however, compared to NaOCl group it was less effective.

## Introduction

Instrumentation of the root canal(s) produces a mud like layer named the smear layer (SL) which contains organic and inorganic components from pulp, dentine, bacteria and their byproducts that occlude the dentinal tubules [1]. There are still some controversies regarding its removal [1, 2], but it seems that advantages of removing SL are more than its disadvantages [3]. SL can prevent the penetration of intracanal medicaments into the radicular dentinal tubules, and might interfere with the sealing ability and adherence of the root filling materials to root canal walls [1, 4].

Laser, ultrasonic and different irrigation solutions are some of the techniques for SL removal [5, 6]; however, the latter is the most common one [1]. The ability to dissolve organic and inorganic components of SL is one of the optimal properties of endodontic irrigants. Moreover, antimicrobial activity and biocompatibility are also desirable [7].

NaOCl is the most common root canal irrigant [8]. However, it has been suggested that it may adversely alter the mechanical properties of the root dentin [9]. Also, it has no remarkable effect on the inorganic (mineral) portion of the SL [1]. A chelating agent such as 17% ethylenediaminetetraacetic acid (EDTA) is recommended for being used accompanied by a proteolytic solution (such as NaOCl) due to its ability to remove the inorganic components of the SL [10, 11]. However, it is stated that EDTA may cause erosion of the root canal dentin in case of prolonged application [12] and it has lower antimicrobial efficacy than NaOCl [13].

**Figure 1:**
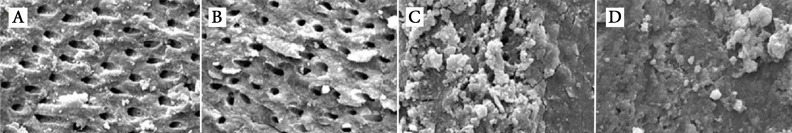
Hülsmann scoring system; *A)* Patent dentinal tubules, *B) *Patent dentinal tubules in more than 50% of the surfaces, *C) *Less than 50% open dentinal tubules; and *D) *No patent dentinal tubules

Application of herbal extracts in dentistry has recently been the center of interest and endodontics is not an exception in this era [14, 15]. The SL removing potential of 2.5% NaOCl, 17% EDTA, extraction of German chamomile and Tea Tree oil has been investigated. Authors suggested that EDTA and NaOCl were the best irrigant followed by German chamomile in second place [15].

Fragaria vesca, commonly called wild (woodland) strawberry has been consumed by humans since the Stone Age. The wild strawberry was first cultivated in ancient Persia where farmers knew the fruit as Toot Farangi. Its seeds were later taken along the Silk Road towards the Far East and to Europe where it was widely cultivated until the 18^th^ century [16]. In medical researches, Fragaria vesca extract (FVE) seems to possess anti-inflammatory and anti-oxidant properties [17, 18] due to its nitric oxide component [18, 19]. 

The effect of various herbal medicaments (except for FVE) on SL removal has been reported earlier [15, 20]. The aim of this study was to evaluate the SL removal potential of FVE in comparison with two common irrigants (5.25% NaOCl, and 17% EDTA) by means of scanning electron microscopy (SEM).

## Materials and Methods

In this analytical-observational *in vitro* study, 40 extracted mandibular and maxillary human teeth were selected. The periapical radiographs confirmed the teeth being caries-free, non-calcified and mature with single root canals within the average length of 21-25 mm without any curvatures. The samples were randomly divided into four groups (*n*=10) based on designated irrigation regiment: Saline (Daroupakhsh, Tehran, Iran) (as positive control), 5.25% NaOCl (Shimin Co., Tehran, Iran) +EDTA (Metabiomed, Chungbuk, Korea) (as negative control), FVE and FVE+EDTA. 

For preparation of FVE, 500 g wild strawberry was grinded by a juicer and admixed with 5000 cc solution of water and ethanol with 50:50 ratio to prepare a 20% concentration. The mixture was transferred to a 500 cc-Erlen tube and mixed for 24 h. The final solution was prepared by using a vacuum machine (Labx, Ontario, Canada) and Whatman filter (Sigma-Aldrich, MO, USA).

After preparing standard access cavities, the working length was measured by using #10 K-files (Dentsply Maillefer, Ballaigues, Switzerland) when their tips were observed under 10× magnification of microscope (Zeiss, Jena, Germany) at anatomical apex. Cleaning and shaping was done with step-back technique with the apical size set at #30 and the canals were flared up to #55. To assure minimum differences in the amount of produced SL, the filing procedure was repeated for 30 times during using each size of instrument. Then 2 cc of each irrigant was derived into the canals between the files by means of 10 cc-syringes and 30-gauge needles (Soha Co., Tehran, Iran). The solutions were maintained for 10 sec in the canals. After cleaning and shaping, final irrigation was followed with 10 cc of the same designated regiment except for the groups in which EDTA was used. Eventually, the canals were flushed with 10 cc of saline.

The teeth were split along their long axis. One half of each specimen was dehydrated and processed according to Kuga *et al.* [21]. The specimens were examined under SEM (LEO1400, Tokyo, Japan), operating at 15 kVp. The evaluation of the remained debris and SL at the coronal, middle and apical thirds of each root canal was done under the magnification of 1500×. 

The specimens were blindly coded and prepared for scoring according to the criteria mentioned by Hülsmann *et al.* [22]: *Score 1;* dentinal tubules completely patent (Figure 1A), *Score 2;* more than 50% of dentinal tubules patent (Figure 1B), *Score 3;* less than 50% of dentinal tubules patent (Figure 1C) and *Score 4;* nearly all of the dentinal tubules occluded with SL (Figure 1D).

In each segment, four sites were demarcated and analyzed and the scores were obtained by three blinded examiners. The collected data were statistically analyzed using the Kruskal-Wallis, Mann Whitney U and Dunnett's tests. The level of significance was set at 0.05.

## Results

Table 1 represents the means of remained SL in each group. Significant differences were observed among the groups (*P*<0.001); NaOCl+EDTA was the most effective regiment for removing the SL followed by FVE+EDTA. FVE alone was significantly more effective than saline (*P*<0.001).

Table 2 shows the pair-wise comparison of each third of the teeth in different groups. To minimize the statistical errors of nonparametric analysis, the Dunnett's test was done. FVE and saline were not effective enough to remove the SL in all of the segments of the root canal. FVE+EDTA showed the greatest efficacy in removing the SL from both coronal and middle thirds. The highest and lowest amounts of remained SL belonged to the first two groups (positive and negative control groups, respectively).

## Discussion

This *in vitro* SEM evaluation revealed that intermittent use of EDTA with either NaOCl or FVE could effectively remove the SL. Based on the results of the present study, the combination of NaOCl+EDTA has a remarkable capability in removing the SL. In the second place, FVE+EDTA left less SL on the dentinal walls.

This *in vitro* SEM evaluation revealed that intermittent use of EDTA with either NaOCl or FVE could effectively remove the SL. Based on the results of the present study, the combination of NaOCl+EDTA has a remarkable capability in removing the SL. In the second place, FVE+EDTA left less SL on the dentinal walls.

Similar results were claimed by Sadr Lahijani *et al.* [15] who compared the amounts of remained SL after irrigation with 2.5% NaOCl, 17% EDTA, extraction of German chamomile and Tea Tree Oil. Their results reflected the efficacy of German chamomile in SL removal that was superior to NaOCl alone but less than NaOCl+EDTA. In another study on herbal extracts, the lowest remnant of SL was found when either Morinda Citrifolia or NaOCl were used in addition to 17% EDTA [20]. In the first mentioned study, the SEM observation and grading of SL removal was done according to the method offered by Hülsmann *et al.* [22]. However, the second study omitted one grade used the following grading system: [without SL (0), incomplete SL removal (1) and complete SL removal (2)]. So, the statistical differences might be due to the different grading systems.

**Table 1:** The mean (SD) of remained smear layer in each group

**Canal region**	**Group **	**Mean (SD)**	***P*** ** value**
**Coronal**	**Saline**	3.9 ± 0.31	<0.001
	**NaOCl+EDTA**	1.8 ± 0.58	
	**FVE**	3.2 ± 0.67	
	**FVE+EDTA**	2.4 ± 0.46	
**Middle**	**Saline**	4.0 ± 0.00	<0.001
	**NaOCl+EDTA**	2.6 ± 0.65	
	**FVE**	3.8 ± 0.33	
	**FVE+EDTA**	2.9 ± 0.69	
**Apical **	**Saline**	4.0 ± 0.00	<0.001
	**NaOCl+EDTA**	2.6 ± 0.47	
	**FVE**	3.8 ± 0.35	
	**FVE+EDTA**	3.4 ± 0.51	

**Table 2.** Pair-wise comparison among different groups

**Groups**	**Group **	**Coronal**	**Middle**	**Apical**
**A/B**	**Saline**	0.001^d^	0.001^d^	0.001^d^
**A/C**	**NaOCl+EDTA**	0.043	0.481	0.280
**A/D**	**FVE**	0.001^d^	0.002^d^	0.023
**B/C**	**FVE+EDTA**	0.001^d^	0.001^d^	0.001^d^
**B/D**	**Saline**	0.029	0.353	0.015
**C/D**	**NaOCl+EDTA**	0.009	0.005^d^	0.123

d statistical significance reported by Dunnett's test (α≤0.007)

Despite the popularity of SEM scoring evaluation, this method has some limitations such as being subjective, less comparative and with a low reproducibility and providing variety of results [23, 24]. By the way, comparison of the SEM scores is still one of the acceptable techniques for evaluating the new solutions, irrigating protocols or even new devices.

The amount of remained SL were higher in apical segment in comparison with the coronal segment in two aforementioned studies [15, 25] which is also confirmed with results of the present study. SL removal might be affected in apical regions due to lesser size of the canal and lower diffusion of irrigations which is mostly caused by insufficient cleaning and shaping, viscosity of the irrigant, needle gauge and experience of the practitioner. Nevertheless, no differences were observed between the apical and coronal regions in another study [20]. 

The present results reflected that the SL remnants were similar in coronal section of the canals in FVE+EDTA group (2.4±0.46) and NaOCl+EDTA group (2.6±0.47); which may suggest that FVE and EDTA would be as much capable in SL removal as NaOCl and EDTA, provided that the solution could diffuse into the apical regions. It has been stated that the required apical flaring for sufficient diffusion of irrigants should be at least #30 [26]. In the present study, the master apical size was set at #30, similar to the method administered by Sadr Lahijani *et al.* [15]. In contrast, Candeiro *et al.* [27] instrumented the root canals up to size #45 to evaluate SL removal ability of apple vinegar. Larger sizes of coronal flaring by using Gates Glidden drills or other instruments, might be helpful for better irrigation but it might weaken the root canal structure due to over instrumentation.

In the present study, the volume of irrigation solution was 2 cc between each instrument and 10 cc of saline was used for final irrigation, which is done according to Sadr Lahijani *et al.* [15]. Also, the concentration of the FVE was 20% in the current study. FVE possesses some anti-proliferative activity that causes a decrease in the weight and volume of contents of granulation tissue during inflammation [28]. As the volume and duration of irrigation have a positive effect on the SL removal ability [29], it is suggested to administer higher volume and longer periods of irrigating with higher concentrations of FVE in future studies.

## Conclusion

FVE with or without EDTA was effective in removing the smear layer, though its efficacy was significantly less than NaOCl+EDTA.
